# CD8^+^ T Lymphocyte and NK Cell Network: Circuitry in the Cytotoxic Domain of Immunity

**DOI:** 10.3389/fimmu.2019.01906

**Published:** 2019-08-13

**Authors:** Roman V. Uzhachenko, Anil Shanker

**Affiliations:** ^1^Department of Biochemistry, Cancer Biology, Neuroscience and Pharmacology, School of Medicine, Meharry Medical College, Nashville, TN, United States; ^2^Host-Tumor Interactions Research Program, Vanderbilt-Ingram Comprehensive Cancer Center, Vanderbilt University School of Medicine, Nashville, TN, United States; ^3^Vanderbilt Center for Immunobiology, Vanderbilt University School of Medicine, Nashville, TN, United States; ^4^Vanderbilt Institute for Infection, Immunology and Inflammation, Vanderbilt University School of Medicine, Nashville, TN, United States

**Keywords:** CD8 T cells (CTL), natural killer cells (NK), lymphocyte crosstalk, immune networks, cytolytic function, effector cooperativity, cancer, immunotherapy

## Abstract

Multiple effector layers in the immune system ensure an optimal temporal and spatial distribution of immune defense. Cytotoxic innate lymphoid natural killers (NK) and adaptive CD8^+^ T lymphocytes (CTL) interact to elicit specific cytolytic outcomes. The CTL carry antigen-specific T cell receptors (TCR) to recognize cognate peptides bound with major histocompatibility complex class-I (MHC-I) or human leukocyte antigen (HLA) molecules on target cells. Upon TCR engagement with MHC-I:peptide at a threshold of avidity, T cell intracellular programs converge into cytolytic activity. By contrast, NK cells lack antigen-specific receptors but express a repertoire of highly polymorphic and polygenic inhibitory and activating receptors that bind various ligands including MHC and like molecules. A highly calibrated maturation enables NK cells to eliminate target cells with lowered or absent MHC-I or induced MHC-I-related molecules while maintaining their tolerance toward self-MHC. Both CTL and mature NK cells undergo membranous reorganization and express various effector molecules to eliminate aberrant cells undergoing a stress of transformation, infection or other pathological noxa. Here, we present the cellular modules that underlie the CTL–NK circuitry to maximize their effector cooperativity against stressed or cancerous cells.

## Introduction

The organization of immune cells into a social network (1) underscores the functional complexity inherent in its design to defend against any pathological noxa. Signals mediated by acting-at-a-distance molecules or juxtaposing intercellular contacts lead to formation of responsive modules necessary for the execution of effector functions. Therefore, organizing functional modules into networking units lets the immune system accomplish a broader task at the level of organism without disturbing organismal homeostasis (2).

Upon a pathological insult, both cellular and humoral immune responses develop through a typical Darwinian selection process. A multitude of cues guide this process: what is the nature of antigen? What is the dose of antigen? Is the antigen self or foreign? What is the appropriate magnitude of response to the antigen? When will the immune contraction phase start? These questions guide the selection of cellular subsets and molecules to launch an appropriate immune response in a universe of diverse antigens. This cannot be tackled by homotypical nodes of lymphocytes acting in isolation. It requires cooperativity from different immune cell subsets.

A repertoire of membrane receptors along with a milieu of intracellular secretory molecules provide input signals to drive various transcriptional master regulators that commit lymphocyte subsets to send a certain array of outputs. Thus, lymphocytes scan the environment for information from other cells as their input and *vice versa*. In other words, immune cell subsets located in proximity can be organized into a unit of mutual receiver-sender modules. In terms of the information theory, this process may be represented as a communication channel for computation. Notably, immune cells respond to instructions from extracellular environment to exercise plasticity in choosing specific cell subsets to launch a dynamic immune response that fits *ad hoc* to input information (2).

In a biological world, different cell types form a stable circuit if they constitutively share information *via* exchange of molecules. For example, the platelet-derived growth factor (PDGF)-secreting macrophages that exclusively express receptors for colony stimulating factor-1 (CSF1) form a stable two-cell circuit with PDGF receptor-expressing fibroblasts, which also supply macrophages with CSF1 (3). Similarly, cancer cells form a reverse Warburg circuit with cancer-associated fibroblasts (CAF) wherein they supply transforming growth factor-β and reactive oxygen species to CAF, triggering their glycolysis, and lactate production. In turn, CAF provide tumor cells with lactate, which is converted into pyruvate and utilized in mitochondrial metabolism necessary for tumor growth and proliferation (4, 5).

In a multicellular network, nodes of individual cells (modules) communicate with each other and act as one regulatory functional unit (analogous to an electrical circuit comprising a transistor, resistor, capacitor, or inductor with logic gates) to manifest the cumulative function of the aggregate of cells. We have called such interconnected modules a circuit in the multicellular network. Here, we discuss the cellular circuitry underlying the two cytotoxic lymphocyte subsets, CD8^+^ T-cells (CTL) and natural killers (NK). Immunosurveillance and cytolytic activity toward transformed or infected cells may benefit from a cross-talk between the CTL and NK cells, which could be recruited at different stages of immune control.

## Cellular Circuitry Underlying CTL Function

CTL recognize their targets *via* a wide repertoire of membrane-expressed T-cell receptors (TCR) present as an octameric complex of variable TCR-α and β chains with three dimeric signaling modules: CD3δ/ε, CD3γ/ε, and CD247ζ/ζ or ζ/η (6). TCR diversity in T-cells is generated by an integration of processes including somatic VDJ recombination, palindromic and random nucleotide additions, and extra-thymic peripheral TCR revision (7, 8). Each TCR complex recognizes a specific MHC/HLA:peptide (antigen) complex cross-presented by the professional antigen-presenting cells (APC) such as dendritic cells (DC), B-cells and macrophages, or presented directly by the target cells.

T-cell interaction with DC represents a classical two-cell circuit wherein an immunological synapse (IS) is maintained by the engagement of multiple pairs of DC-expressed ligands with T-cell-expressed receptors such as ICAM-1 (CD54):LFA-1 (CD11a-CD18 heterodimer), CD80/CD86:CD28, MHC-peptide:TCR, CD40:CD40L (CD154) (9). It is important to note that CD40:CD40L binding ushers DC to secrete IL-12, an instructive cytokine for T-helper-1 (Th1) development, whereas MHC-peptide:TCR and CD80/CD86:CD28 interactions trigger production of IL-2, a proliferative cytokine, by T-cell subsets (10). Moreover, by a prolonged IS, DC and CTL provide each other with survival signals. The costimulatory molecule CD28 engagement on CTL activates the PI3K/Akt survival pathway, and prevent anergy (hyporesponsiveness) by upregulating Bcl-xL and downregulating CD95L (10). The growing list of costimulatory receptors expressed on T-cells includes 4-1BB (CD137), OX40 (CD134), TNFRSF7 (CD27), ICOS (CD278), TNFRSF8 (CD30), LFA-2 (CD2), DNAM-1 (CD226), and NKG2D (CD314) among others (11–13). DC survival is associated with the stimulation of CD40:CD40L axis (9). Lately, the importance of specific Notch receptor-ligand interactions has also been demonstrated in the antitumor DC–CTL network (14–16).

For CTL activation, a three-cell circuitry has been proposed. Initial model assumed that a single DC can bind both CD4^+^ T and CD8^+^ T cells through the expression of MHC-II and MHC-I molecules, respectively. In this three-cell interaction, CD4^+^ T while synapsed with DC supply IL-2 whereas DC provide co-stimulatory signals to CD8^+^ T cells (17). Later, this model was modified to an alternative view in which DC sequentially interact with CD4^+^ T and CD8^+^ T cells, thus forming temporary bridges between the two T-cell subsets. After dissociation from the DC:CD4^+^T licensing coupling *via* CD40:CD40L interaction, the same DC presents antigen to CD8^+^ T-cells in a dynamic DC:CD4^+^T:CD8^+^T serial interaction (18). Further, trogocytosis (intercellular transfer of membrane proteins) observed between DC and CD4^+^ T supports the dynamic three-cell serial interaction exhibited by CD4^+^ T acquiring MHC-I:peptide complexes from DC to present to CD8^+^ T with concurrent provisions of instructive cytokines (IL-2, IL-12, etc.) and co-stimulation (19). Currently accepted dynamic three-cell interaction model proposes that during DC:CD4^+^T interaction, DC become licensed whereas CD4^+^ T acquire MHC:peptide complexes and transform into primed CD4^+^T:DC clusters. CD8^+^ T-cells then interact with CD4^+^T:DC cluster or licensed DC alone (20). Thus, trogocytosis and expression of wide variety of costimulatory molecules allow CD8^+^ T-cells to flexibly find their interaction partners, and activate specific transcriptional programs to support the expression of proteins responsible for effector function. It is these differentiated mature CTL in the lymph nodes, which extravasate into the area of infected cells or tumor and re-engage with the cognate MHC-I:peptide complexes to execute their effector programs. Since secretion of a large number of apoptosis-triggering molecules may be harmful for surrounding tissue, a synaptic contact with the tumor or infected cells that allows a polarized or membranous-vesicular or nanotube-guided delivery of cytolytic granules will avoid bystander off-target CTL cytotoxicity.

## Cellular Circuitry Underlying NK Function

NK are enigmatic in that they display intrinsic (“natural”) ability to lyse tumor, infected or stressed cells without prior priming (21). Although initially considered an artifact, it became obvious by the 1970s that NK represent a population distinct from antigen-specific T-cells (22–25). Yet, their cytolytic activity continued to be overshadowed by large granular cytotoxic lymphocytes (26). Recent developments in transcriptomics finally defined their molecular identity as innate cytotoxic lymphocytes and established their distinct yet overlapping patterns with CD8^+^ CTL (27–29). They have lately garnered interest due to their “on demand” NK-poiesis coordinated in space and time (30).

Unlike CTL, where diversity lies in rearranged TCRs specific to antigens, and tolerance to self is achieved by selective survival of developing thymocytes, NK express a diverse repertoire of germ-line encoded activating and inhibitory receptors to generate diversity and maintain tolerance. NK receptors belong to either the type-1 transmembrane proteins of the immunoglobulin superfamily (e.g., activating natural cytotoxicity receptors, NCRs), the immunoglobulin-like superfamily (e.g., human killer-cell immunoglobulin-like receptors, KIRs), or the C-type lectin-like receptor superfamily (e.g., CD94/NKG2A heterodimer and the multigenic murine Ly49). Binding partners for these receptors are classical or non-classical MHC-I molecules or MHC-I laden with foreign peptide or pathogen-encoded molecules. Although initial expression of inhibitory and activating receptors on NK appears to be stochastic, an education process involving MHC-I alleles expressed by the host tissue determines the final repertoire of NK receptor expression (31). Several models of NK education have been proposed to balance the stimulatory and inhibitory signals and calibrate their reactive potential.

The “*licensing and arming*” models assume that NK education is dependent on the phosphorylation of immunoreceptor tyrosine-based inhibitory motif (ITIM) in intracellular domains of NK-inhibitory receptors upon binding with classical MHC-I. Subsequent downstream signaling triggers NK cell acquisition of effector functions (32). A “*disarming model*” postulates that in the absence of inhibitory signals NK stay in a sustained state of activation but they become hyporesponsive upon engagement with cognate inhibitory self-MHC class-I ligands (33). “*Rheostat model*” describes NK education as a process where magnitude of the integrated inputs from different inhibitory signals (MHC-I and non-MHC-I ligands) translate into the strength of effector output (34). “*Tuning model*” proposes a refinement of NK responsiveness to sudden modifications within the host environment in line with the discontinuity theory of immunity (35, 36). The balancing of the stimulatory and inhibitory signaling through multicellular receptor:ligand engagements calibrates NK activity in conjunction with the target cell recognition through lowered or absent self-MHC or HLA expression termed “*missing-self* or *induced-self recognition*” of aberrant cells undergoing transformation, infection or other pathology. Cellular stress induces ligands for NK-activating receptors, such as NKG2D, namely MICA, MICB, and UL16-binding proteins (ULBP), all of which are MHC-1-related molecules. Thus, NK display transcriptional pre-primed state, which may allow them to stay “alert” and mount effector responses rapidly after encountering targets.

## Bidirectional Circuitry Between CTL and NK

Overlapping cytolytic abilities of CTL and NK warrant a close regulated collaboration. NK-expression of a wide range of chemotactic, synaptic, effector, and regulatory molecules allow NK to form multiple contacts with various cells, which also would affect CTL (37). NK express CXCR3 chemokine receptor directing them to lymph nodes, where they interact with DC and affect their maturation (38, 39). As mentioned above, DC form dynamic three-cell interaction circuit with CD4^+^ T and CD8^+^ T cells. NK would thus modify the dynamics of DC–CD4^+^T–CD8^+^T circuitry *via* a bi-directional cytokine exchange and cell-to-cell contacts. DC secrete IL-12, type-I IFN, TNF-α, and express MIC-A and B ligands for the NK-activating receptor NKG2D. In turn, NK support DC maturation through IFN-γ and TNF-α production. This promotes Th1 polarization and CTL responses (37, 40, 41). By NK-mediated target lysis, the content of antigens is increased for DC presentation to CTL (42). NK can also selectively kill DC based on their maturity status, and thus affect the strength of CTL response. It is known that mature DC express inhibitory ligands for KIR whereas immature DC lack these ligands, making the latter susceptible to NK-mediated lysis (39, 40).

Further, NK may acquire MHC-II and other molecules through trogocytosis and compete with DC for interaction with CD4^+^ T-cells. Such NK do not activate T-cells, rather suppress them (43). Besides, activated T-cells often up-regulate ligands for NKG2D and DNAM-1, which make them prone to lysis by NK. Lysis of activated CD4^+^ T-cells will compromise T-cell help to CD8^+^ T-cells (40). Also, NK secrete IL-10, which inhibits CD8^+^ T-cell proliferation (44). At the same time, to resist NK attacks, T-cells employ mechanisms such as express IFN-α receptor, upregulate MHC-I molecules (KIR ligands) and CD48 (2B4 ligand) or down-regulate NCR1 ligands (45). It has been hypothesized that potentially autoimmune CTLs (deprived of essential signals, such as type-I IFN) are eliminated by NK (40).

Moreover, CD8^+^ CTL can also affect NK activation. Studies have demonstrated that CTL can activate intratumoral NK cells (46–48). This teamwork of CD8^+^ T and NK prevents the development of antigen-deficient tumor escape variants. No role of a bystander CTL lysis of antigen-deficient tumor cells or tumor stroma following possible uptake of antigen from the lysed antigen-expressing tumor cells was found (49, 50). Rather, antitumor NK activity was observed when CTL were present in vicinity of NK. Gene profiling of NK from the tumor where antigen-specific CTL were present showed a strong expression of effector (*Gzma, Gzmb, Prf1*, and *4.1BB*), tissue–migratory (*Gpr33* and *Ccr5*), and signaling (*Ifngr, Klhdc2, Eif3s6, Map3k6, Tnfrsf1b, Icos, Ly49G*, and *Nmi*) transcripts. This suggested that tumor-infiltrating NK gene expression program was influenced by locally present CTL. Separation of antigen-deficient variants (NK targets) from the antigen-expressing tumors (CTL targets) prevented CD8^+^ T-cell help to NK (47). Cooperative CTL–NK interaction in tumor rejection especially under conditions of limited TCR diversity (46), involving NKG2D-mediated mechanisms, has been observed in multiple tumor models (51–56).

**Figure 1:**
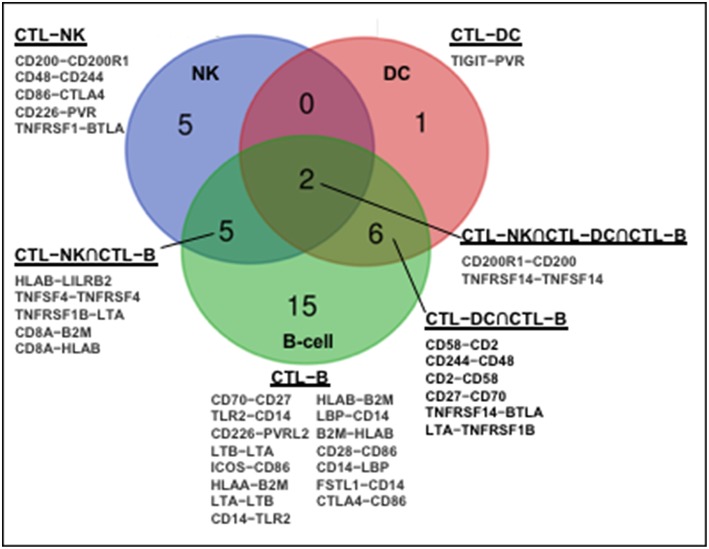
Molecular partners of functional immune network formed by human CD8^+^CTL with NK, DC, and B cells. Human immune network proteomics public resource (http://www.immprot.org/) containing a depth of >10,000 proteins was analyzed to delineate molecular partners for human CD8^+^T interaction with NK and two classical antigen-presenting cells, dendritic cells (DC), and B-cells. The following cell subsets were considered: (1) activated CD56^bright^ and CD56^dim^ NK-cells; (2) naïve central-memory, effector-memory and EMRA CD8^+^T-cells; (3) activated myeloid and plasmacytoid DC; and (4) naïve and memory B-cells. Molecular partners in intercellular contacts formed by CD8^+^T-cells and NK (blue, CD8^+^CTL–NK), dendritic (red, CD8^+^CTL–DC), or B (green, CD8^+^CTL–B) cells were compared. Venn diagram represents common Boolean molecular couples (cross-sections, merged colors) as well as unique intermolecular interactions (single color) based on STRING database score > 0.7 for known and predicted protein–protein interactions.

The human immune network proteomics resource containing a depth of >10,000 proteins (1) supports profound intercellular circuitry based on the sender (cytokines, membrane ligands) and receiver (receptors) molecules on different immune cells. Database analysis demonstrates that CD8^+^CTL–NK interface occupies intermediate position between CTL–DC and CTL–B-cell in terms of the number of participating immune stimulatory and inhibitory molecules (Figure 1). B-cells and DC appear classical interaction partners for CD8^+^ T-cells for antigen presentation. A dynamic regulation within CTL–NK circuit is pointed by a spectrum of surface co-stimulatory and co-inhibitory molecules, such as ligand adaptor SLAM-associated protein (SAP), CD48, on CTL with receptor 2B4/CD244 on NK. This molecular interaction may protect CTL from NK lytic attack as well as inhibit or activate NK depending on the intracellular concentrations of CD48 and CD244 (57). Analysis also indicates that NK express stimulatory molecules from the Nectin-like family (CD155/poliovirus receptor) with potential to interact with CD266 (DNAM-1) on CTL. Further, expression of lymphotoxin-A and tumor necrosis factor superfamily-14 (TNFSF14/LIGHT) by NK and complementary TNFRSF1B and TNFRSF14/HVEM (herpes virus entry mediator) by CTL may serve as a substrate for CTL–NK compartmentalization in tumors or tumor-draining lymph nodes. These receptor-ligand couples are involved in the formation of secondary and tertiary lymphoid organs, ectopic lymphoid tissues necessary for antitumor T-cell immunity (58, 59). Finally, the expression of CD8A molecule by CTL and β2-microglobulin and HLA-B molecules by NK correlated with NK antigen presentation. APC-like properties were demonstrated for porcine NK cells (60). Notably, there is no molecular partner exclusive to the circuitry among CTL, NK, and DC (Figure 1).

The lytic function of CTL–NK effector circuit is fine-tuned by checkpoint inhibitory molecules from B7/CD28 family, CTLA-4 and BTLA, expressed on NK partnered with CD86 and TNFRSF1, respectively, expressed on CTL. CTLA-4 was found to be up-regulated with CD28 after NK stimulation with IL-2 while ligation of CTLA-4 with B7 molecules inhibited IFNγ production (61). Negative effect of blocking BTLA on NK has been reported (62). Further, while CTL express inhibitory ILT/CD85 member, LILRB2, NK express its interaction partner HLA-B. The up-regulation of LILRB2/ILT4 on NK may increase their activation threshold (63). Also, CD200-CD200R1 expressed by CTL and NK, respectively, may represent another inhibitory coupling controlling CTL–NK lytic circuitry (Figure 1). Suppressive CD200R1 cross-linking on NK was demonstrated for acute myeloid leukemia as one escape mechanism for tumor growth (64).

## Conclusions

Formation of negative and positive feedback loops in the CTL–NK lytic circuitry suggests that multiple functional modules are responsible for sensing and killing target cells undergoing cellular stress of infection, transformation or other pathological noxa. As we proposed earlier (54), antigen-specific adaptive immune cells provide tissue specificity and guide recruitment of innate effector cells to the site of tumor or other pathological insults. While the CTL subset, scans tissue to lyse targets in an antigen-specific manner, the NK subset recognizes and eliminates antigen-escape variants under the antigen-selective pressure of CTL.

The topology and the spectrum of lytic circuitry appears to be defined by the nature and dose of antigens and the context of tissue microenvironment. Thus, many bacterial or acute viral antigens elicit B-cell response and require the presence of Th2 cells to support antibody production. A persistent/chronic viral infection involves intracellular antigen processing and cross-presentation by DC and differentiation of CTL responses. However, availability of immune cells and their maturation/priming/activation status is important for the outcome. Recently, CD103^+^ DC expressing basic leucine zipper ATF-like transcription factor-3 (Batf3) were found necessary for recruiting and activating CD8^+^ T-cells in tumors (65, 66). Moreover, when DC decline in number, B-cells take over their role (67). Indeed, CD11b^+^ B-cells with potent APC function border T-B cellular area in spleens (68). This is also supported by the observation that CD20^+^ B-cells are co-present with CTL in ovarian cancer (69). Apparent proximity of CTL and NK inside solid tumors (47) offer multiple opportunities for their interactions. Accordingly, DC, CTL, and NK form a cellular network of functional circuitry, characterized by redundancy and degeneracy necessary for robust network properties. Such topological organization has obvious advantages for system flexibility: deficiency in individual elements of network will rearrange connections between remaining elements, thereby increasing functional robustness to perturbations. The proposition goes in line with the concept of co-respondence proposed for the immune system (2).

From an evolutionary viewpoint, activation of NK by CTL to eradicate aberrant or tumor cells may be considered a bet hedging strategy. Bet hedging is the ability of cells or organisms to diversify their phenotypes to increase future fitness at the expense of benefits in current situation (70). The latter is common in the prokaryotic and eukaryotic worlds. Antibiotic resistance in *Mycobacterium tuberculosis* (71) or cannibalism in *Bacillus subtilis* during spore formation (72) are examples of bet hedging in prokaryotes. In eukaryotes, a classical bet hedging is production of different size eggs by animals in a clutch (70). Multiple types of drug resistance and cellular heterogeneity in individual tumors are also indicative of increased tumor fitness (73, 74).

Thus, the CTL–NK circuitry may indicate a provision by which CTL curb the expansion of targets and their escape variants by recruiting NK cells. Examples are prevalent where impaired CTL–NK communication or lack of either partner results in the failure of CTL–NK circuitry and leads to disease progression. In lymphocytic choriomeningitis infection, at high viral doses, NK prevent excessive CTL response whereas at suboptimal lower doses, NK facilitate CTL-mediated lethality. Hence, a perturbed or imbalanced CTL–NK axis can cause severe immunopathology (75, 76). Consequently, nature appears to have evolved another layer of immune control. Immune cells are found in the brain (77–79), with evidence of modulation of anti-bacterial (80) and anti-tumor (81) immune responses by the brain's reward system. In this context, dynamics of CTL and NK cell circuitry and their lytic capacity need to be optimized with supplementation from a neurostimulatory mood-enhancing neuronal circuitry in the central nervous system (81) to develop effective immunotherapies capable of out-pacing infections, cancer or other pathologies.

## Data Availability

Publicly available datasets were analyzed in this study. These data can be found here: http://www.immprot.org/.

## Author Contributions

RU performed *in silico* analysis of immune network interactions (http://www.immprot.org/). AS and RU conceived, designed, and wrote the manuscript. Both authors read and approved the final manuscript.

### Conflict of Interest Statement

The authors declare that the research was conducted in the absence of any commercial or financial relationships that could be construed as a potential conflict of interest.
